# Predicting depression among rural and urban disabled elderly in China using a random forest classifier

**DOI:** 10.1186/s12888-022-03742-4

**Published:** 2022-02-15

**Authors:** Yu Xin, Xiaohui Ren

**Affiliations:** West China School of Public Health and West China Fourth Hospital, Sichuan University, Chengdu, Sichuan China

**Keywords:** Disabled elderly, Depression, Machine learning, Random forest

## Abstract

With global aging, the number of elderly with physical disabilities is also increasing. Compared with the ordinary elderly, the elderly who lose their independence are more likely to have the symptoms of depression. Reducing depression may help to alleviate the disability process of those who find themselves in the disabled stages. Therefore, the purpose of this study is to explore the predictive effects of demographic characteristics, health behavior, health status, family relations, social relations, and subjective attitude on depression in rural and urban disabled elderly to improve early depression symptom recognition.

A total of 1460 older adults aged 60 and disabled were selected from China Family Panel Studies (CFPS). Depression was assessed according to The Center for Epidemiologic Studies Depression Scale (CES-D). This paper used the random forest classifier to predict the depression of the disabled elderly from six aspects: demographic characteristics, health status, health behavior, family relationship, and social relationship. The prediction model was established based on 70% of the training set and 30% of the test set. The depression rate of rural disabled elderly was 57.67%, and that of urban disabled elderly was 44.59%. The mean values of the 10-k cross-validated results were 0.71 in rural areas and 0.70 in urban areas. AUC:0.71, specificity: 65.3%, sensitivity: 80.6% for rural disabled elderly with depression; AUC:0.78, specificity: 78.1%, sensitivity: 64.2% for urban disabled elderly with depression, respectively. There are apparent differences in the top ten predictors between rural and urban disabled elderly. The common predictors were self-rated health, changing in perceived health, disease or accidence experience within the past 2 weeks, life satisfaction, trusting people, BMI, and having trust in the future. Non-common predictors were chronic diseases, neighborly relations, total medical expenses within 1 year, community emotion, sleep duration, and family per capita income. Using random forest data to predict the depression of the disabled elderly may lead to early detection of depression.

## Background

Aging is a challenge for all countries, and the crisis is even more significant in low- and middle-income countries [1]. Increasing incidences of physical disability are associated with aging, and that significant heterogeneity exists among older populations [2]. Disability refers to the difficulty or inability to perform tasks essential to everyday life, affecting social roles, and maybe a result of physical, emotional, cognitive, or sensory limitations [3]. The main pathway of disability includes four successive stages: pathology (the existence of disease/injury), impairments (dysfunction / structural abnormality), functional limitation (basic physical/mental activity limitation), and disability (difficulty doing activities of daily life, ADL) [4]. In the process of disability, depression plays an accelerating role, especially in the early and late stages of disability. Reducing depression may help alleviate the disability process of those who find themselves in the above stages [5]. Compared with the ordinary elderly, the disabled elderly are more likely to have the symptoms of depression [6, 7]. Özlem and Ünsal found that the incidence of depression was 57.8% (*n* = 201) in disabled people [8]. Therefore, the depression of the disabled elderly needs social attention.

There also have been some previous studies on the influencing factors of depression in disabled adults [8]. In Özlem’s cross-sectional study, depression in disabled adults was associated with demographic characteristics (married people tend to develop depression), health behaviors (smoking and drinking tend to develop depression), and family status (three or more children are more likely to develop depression) [9]. In the factors influencing the depression-exit of the disabled, JunSu found socioeconomic state-related factors (gender, marital status, and the regional location), psycho-social characteristics related factors (self-esteem and the satisfaction about leisure and recreation), and disability-health related factors affect depression withdrawal of the disabled [10].

Previous studies have shown differences in the risk of depression between urban and rural elderly, which may be due to the differences in the living environment between urban and rural elderly [8]. Among urban and rural Chinese older adults, Yu found that the prevalence rates of depression in urban and rural areas were 16.4 and 30.0% [11]. However, the dynamics of depression affecting urban and rural people are complex and may vary with different health status, populations, and national backgrounds [8, 12].

Machine learning is increasingly used in depression [13–15]. Compared with human experts, the amount of data, computational complexity, and storage capacity of medical decision support systems are relatively high [16]. Random forest is a flexible and easy-to-use machine learning algorithm. It includes a random forest classifier and random forest regression. Previous studies have applied a random forest classifier to predict depression in different populations. In the prediction of depression in nursing staff of patients with Alzheimer’s disease, Byeon showed that gender, subjective health status, disease or accidence experience within the past 2 weeks, the frequency of meeting a relative, economic activity, and monthly mean household income were the significant predictors for the depression of caregivers [17]. Gokten and Uyulan used a random forest classifier to predict the development of depression and post-traumatic stress disorder development in sexually abused children. They found that the most important feature of the prediction model is time after abuse, type of abuse, and smoking [14]. Due to the different research objects, there are great differences in the important predictors.

However, to the best of our knowledge, seldom studies built a machine learning-based model for predicting the onset of depression among disabled elderly, and there is rarely research to indicate the difference of influencing factors of depression symptoms between urban and rural elderly and the extent to influencing factors of the depressive symptom disparity.

## Methods

### Data collection

The data were derived from the China Family Panel Studies (CFPS). CFPS is a biennial longitudinal survey conducted by the Institution of Social Science Survey at Peking University. This investigation launched in 2010, with five waves of publicly released datasets comprising 2010, 2012, 2014, 2016, and 2018. The samples covered 25 provinces, accounting for 95% of the total population of China. The contents of CFPS are rather typical, covering the demographics, socioeconomic condition, education, and health of respondents. According to the research purpose, we chose the data in 2016 because the survey before 2016 did not include the depression scale.

**Fig. 1:**
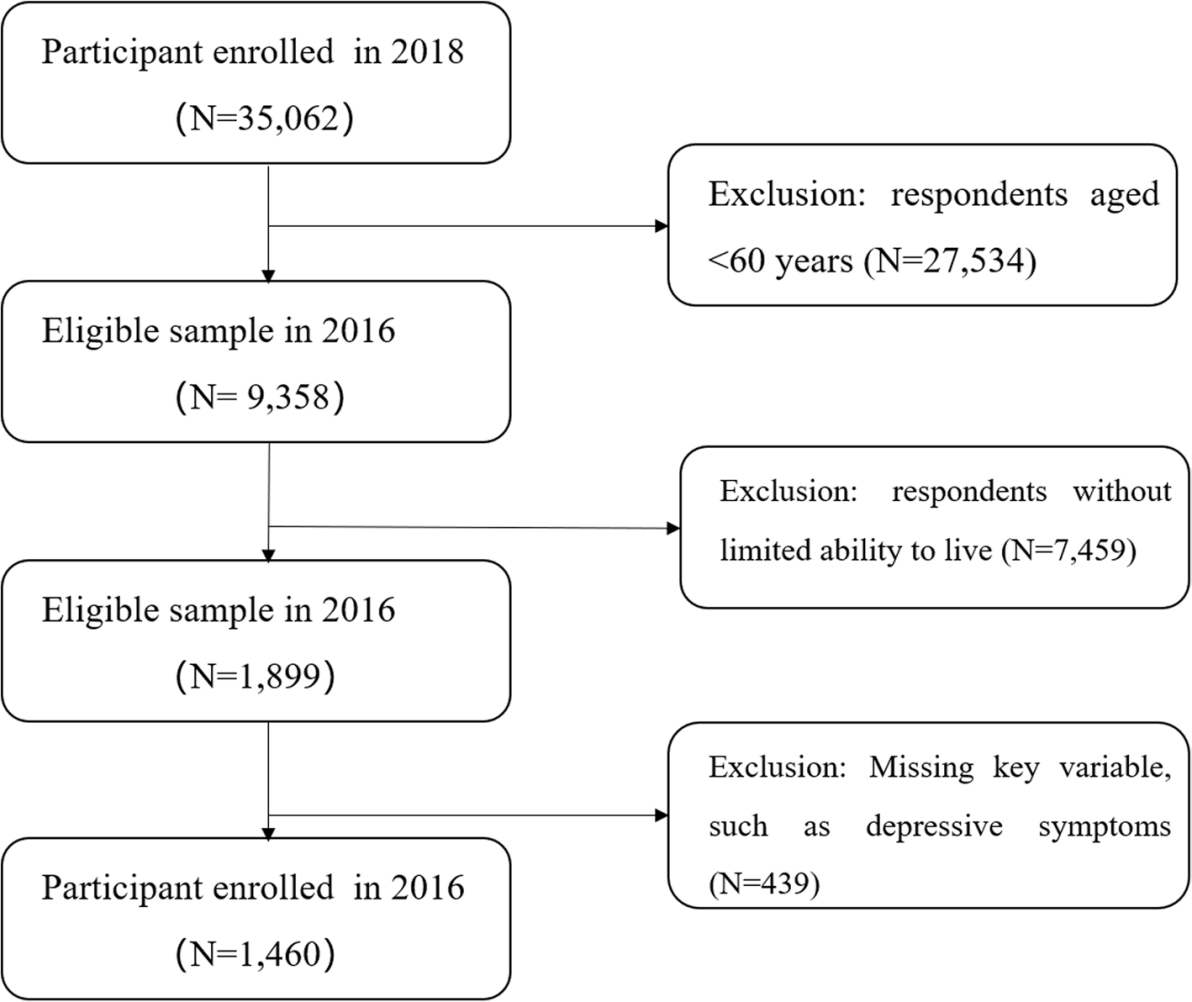
Flowchart of participant selection

The object of this study is elderly with limited ability to live. The items for measuring disability were based on the IADL scale [18]: going to outdoor activities, dining, kitchen activities, public transportation, shopping, cleaning, and laundry. If any of the seven activities cannot be completed independently, it is defined as a disability in this paper. Finally, 1460 participants met the requirements, including 841 rural elderly and 619 urban elderly. The sample selection flowchart is shown in Fig. 1.

### Study design and variables

In the 2016 CFPS questionnaire, depressive symptoms were measured by The Center for Epidemiologic Studies Depression Scale (CES-D). Participants were asked to assess how often they experienced happiness, loneliness, and hope in the previous week. This scale allows respondents to self-rate their degree of experience using a four-point scale: “rarely or never (less than 1 day)”, “not too often (1–2 days)”, “sometimes or half the time (3–4 days)”, and “most of the time (5–7 days)”. The responses for the items of negative feelings were assigned to an index value of 0, 1, 2, and 3, and those to positive feelings were assigned as 3, 2, 1, and 0. The total score ranged from 0 to 60. More than 16 scores of adults were positive screening of depression [19]. CES-D 20 and CES-D 8 questionnaires were used in the survey, respectively, among which 20% of the respondents used CES-D 20 and the remaining 80% used CES-D 8. The scales used by the respondents were randomly assigned. The score of CES-D 8 was transformed into that of CES-D 20 by equal percentile transformation. After transformation, the percentile distribution of CES-D 20 scores of the two groups was similar and had almost the same mean, standard deviation, kurtosis, and skewness. The output variable is CES-D 20 result, classified as “1” depression and “0” non-depression.

The predictors are divided into six categories, including demographic characteristics (gender, age, marital status, years of education, family per capita income, and urban and rural), health behavior (smoking, drinking more than three times a week, sleep duration, and regular exercise), health status (chronic disease, BMI, disease or accidence experience within the past 2 weeks, hospitalization within 1 year, total medical expenses within 1 year, self-rated health, and changing in perceived health), family relations (number of family members, number of children, close to children, receiving financial assistance from children, and weekly family dinner frequency), social relations (neighborhood help, neighborhood relationship, community emotion, participating organizations, and trusting people), and subjective attitude (life satisfaction and having trust in the future).

### Statistical analysis

Random forest (RF) algorithm is an integrated model that uses various models to evaluate the response and is designed to solve the classification and regression problems. RF algorithm can be applied to continuous data sets and classification data sets. This paper uses a Random Forest Classifier (RFC), consisting of many individual decision trees that operate as an ensemble. Each tree in the random forest is predicted and voted, with the most voted class becoming predictive of the entire model. Compared with a single model, one of the advantages of RFC is that each tree classifier is like a team member, and all members work together to obtain the final prediction, which performs better than when using a single decision tree. RFC is suitable for binary classification. It can cope with a dataset where the number of variables exceeds the number of observations and handle the dataset with a mixture of continuous and categorical predictors. RFC also has good noise resistance, can process high latitude data without feature selection, process various kinds of data, and get the order of variable importance. The data were randomly divided into two sets in the rural and urban model: training set (70% of the sample) and testing set (30% of the sample).

We used generalization error and model complexity to adjust the parameters of the RFC, developed to avoid overfitting by simplifying the decision tree by removing terminal nodes. As a result of this process, the predictive power of the model could be enhanced. So, we identified hyper-parameters used commonly in RFC [20]: (1) n-estimators (number of trees in the forest); (2) max depth (maximum depth of the tree); (3) min_samples_split (minimum number of data points in a node before the node is split); and (4) min_samples_leaf (minimum number of data points allowed in a leaf node). The learning curve was used to evaluate two sets of hyper-parameters to optimize the algorithm’s performance. In the context of machine learning, learning curves are used to select the optimal combination of parameters. Table 1 shows the results for rural and urban areas.

**Table 1:** Random forest model and training parameters

Parameter	Value(rural)	Value(urban)
n_estimator	128	190
min_samples_split	16	16
min_samples_leaf	8	8
max_depth	7	7

## Results

### Participant characteristics

A total of 1460 individuals were included in the analysis. The prevalence of depression varied substantially between urban and rural older adults, and the prevalence of depression of urban, rural, and all older adults were 44.59, 57.67, and 52.12%, respectively. Table 2 summarizes the demographic characteristics, health behavior, health status, family relations, social relations, and subjective attitudes of the disabled elderly in rural and urban areas. There is statistical significance in the years of education, family per capita income, regular exercise, BMI, disease or accidence experience within the past 2 weeks, hospitalization within 1 year, total medical expenses within 1 year, self-rated health, changing in perceived health, number of family members, number of children, receiving financial assistance from children, community emotion, participating organizations, life satisfaction, having trust in the future and depression of the disabled elderly between rural and urban areas. Therefore, we consider the construction of depression prediction models for the disabled elderly in urban and rural areas.

**Table 2:** Sociodemographic data and characteristics of rural and urban disabled elderly

Variables	Rural				Urban				*P* value_1_
	Non-depression	Depression	*P* value	Sum	Non-depression	Depression	*P* value	Sum	
**Demographical factors**									
Gender									0.636
Male	164(46.07)	187(38.56)	0.029	351(41.74)	153(44.61)	113(40.94)	0.360	266(42.97)	
Female	192(53.93)	298(61.44)		490(58.26)	190(55.39)	163(59.06)		353(57.03)	
Age	71.35(0.38)	70.63(0.31)	0.0719	70.93(0.24)	72.51(0.43)	72.44(0.47)	0.911	72.48(0.32)	
Marital status									0.179
Married	262(73.60)	311(64.12)	0.004	573(68.13)	251(73.18)	191(69.20)	0.277	442(71.41)	
Unmarried/divorced/widowed	94(26.40)	174(35.88)		268(31.87)	92(26.82)	85(30.80)		177(28.59)	
Years of education									0.000
0	261(73.31)	384(79.18)	0.001	654(76.69)	218(63.56)	175(63.41)	0.992	393(63.49)	
1 ~ 12	61(17.13)	43(8.87)		104(12.37)	90(26.24)	72(26.09)		162(26.17)	
> 12	34(9.55)	58(11.96)		92(10.92)	35(10.20)	29(10.51)		64(10.34)	
Ln (Family per capita income)	8.83(0.05)	8.67(0.04)	0.0218	13,721.75(2462.48)	9.63(0.06)	9.39(0.06)	0.004	24,088.76(1953.38)	0.001
**Health behavior**									
Smoking									0.352
Yes	86(24.16)	98(20.21)	0.171	184(21.88)	71(20.70)	52(18.84)	0.564	123(19.87)	
No	270(75.84)	387(79.79)		657(78.12)	272(79.30)	224(81.16)		496(80.13)	
Drinking more than three times a week									0.828
Yes	39(10.96)	37(7.63)	0.096	76(9.04)	39(11.37)	19(6.88)	0.057	58(9.37)	
No	317(89.04)	448(92.37)		765(90.96)	304(88.63)	257(993.12)		561(90.63)	
Sleep duration									0.064
< 6 h	34(9.55)	97(20.00)	0.000	131(15.58)	36(10.50)	80(28.99)	0.000	116(18.74)	
6-8 h	110(30.90)	131(27.01)		241(28.66)	111(32.36)	84(30.43)		195(31.50)	
> 8	212(59.55)	257(52.99)		469(55.77)	196(57.14)	112(40.58)		308(49.76)	
Regular exercise									0.000
Yes	132(37.08)	149(30.72)	0.053	281(33.41)	167(48.69)	112(40.58)	0.044	279(45.07)	
No	224(62.92)	336(69.28)		560(66.59)	176(51.31)	164(59.42)		340(54.93)	
**Health status**									
Chronic disease									0.147
Yes	94(26.40)	229(47.22)	0.000	323(38.41)	125(36.44)	136(49.28)	0.001	261(42.16)	
No	262(73.60)	256(52.78)		518(61.59)	218(63.56)	140(50.72)		358(57.84)	
BMI	21.93(0.23)	21.29(0.21)	0.0424	21.57(0.15)	23.04(0.23)	22.32(0.26)	0.037	22.73(0.17)	0.000
Disease or accidence experience within the past 2 weeks									0.005
Yes	147(41.29)	335(69.07)	0.000	482(57.31)	133(38.78)	176(63.77)	0.000	309(49.92)	
No	209(58.71)	150(30.93)		359(42.69)	210(61.22)	100(36.23)		310(50.08)	
Hospitalization within 1 year									0.005
Yes	82(23.03)	176(36.29)	0.000	258(30.68)	114(33.24)	119(43.12)	0.012	233(37.64)	
No	274(76.97)	309(63.71)		583(69.32)	229(66.76)	157(56.88)		386(62.36)	
Ln (Total medical expenses within 1 year)	7.32(0.09)	7.92(0.08)	0.000	6730.30(538.75)	8.30(0.01)	8.55(0.10)	0.081	12,486.44(1189.13)	0.000
Self-rated health									0.023
Poor	127(35.67)	316(65.15)	0.000	443(52.68)	108(31.49)	170(61.59)	0.000	278(44.91)	
Fair	84(23.60)	82(16.91)		166(19.74)	92(26.82)	66(23.91)		158(25.53)	
Good	90(25.28)	68(14.02)		158(18.79)	97(28.28)	32(11.59)		129(20.84)	
Very good	36(10.11)	9(1.86)		45(5.35)	31(9.04)	6(2.17)		37(5.98)	
Excellent	19(5.34)	10(2.06)		39(3.45)	15(4.37)	2(0.72)		17(2.75)	
Changing in perceived health									0.006
Better	23(6.46)	34(7.01)	0.000	57(6.78)	32(9.33)	10(3.62)	0.000	42(6.79)	
Unchanged	133(37.36)	78(16.08)		211(25.09)	140(40.82)	62(22.46)		202(32.63)	
Worse	200(56.18)	373(76.91)		573(68.13)	171(49.85)	204(73.91)		375(60.58)	
**Family relations**									
Number of family members									0.000
< 3	123(34.55)	181(37.32)	0.711	128(15.22)	123(35.86)	114(41.30)	0.383	133(21.49)	
3–5	130(36.52)	170(35.05)		394(46.85)	152(44.31)	112(40.58)		332(53.63)	
> 5	103(28.93)	134(27.63)		319(37.93)	68(19.83)	50(18.12)		154(24.88)	
Number of children									0.000
< 3	52(14.61)	76(15.67)	0.758	304(36.15)	71(20.70)	62(22.46)	0.714	237(38.29)	
3–6	172(48.31)	222(45.77)		300(35.67)	189(55.10)	143(51.81)		264(42.65)	
> 6	132(37.08)	187(38.56)		237(28.18)	83(24.20)	71(25.72)		118(19.06)	
Closing to children									0.921
Yes	302(84.83)	398(82.06)	0.288	700(83.23)	299(87.17)	215(77.90)	0.002	514(83.04)	
No	54(15.17)	87(17.94)		141(16.77)	44(12.83)	61(22.10)		105(16.96)	
Receiving financial assistance from children									0.000
Yes	197(55.34)	181(37.32)	0.032	501(59.57)	149(43.44)	126(45.65)	0.582	344(55.57)	
No	159(44.66)	304(62.68)		340(40.43)	194(56.56)	150(54.35)		275(44.43)	
Weekly family dinner									0.393
Seven times	319(89.61)	405(83.51)	0.012	724(86.09)	301(87.76)	222(80.43)	0.012	523(84.49)	
Less than seven times	37(10.39)	80(16.49)		117(13.91)	42(12.24)	54(19.57)		96(15.51)	
**Social relations**									
Neighborhood help	1.70(0.05)	1.41(0.05)	0.000	1.58(0.04)	1.48(0.05)	1.66(0.06)	0.013	1.56(0.04)	0.615
Neighborhood relationship	2.25(0.38)	1.88(0.04)	0.000	2.10(0.03)	2.04(0.04)	2.21(0.05)	0.020	2.14(0.03)	0.196
Community emotion	2.01(0.04)	1.73(0.4)	0.000	1.89(0.03)	1.87(0.05)	2.18(0.06)	0.000	2.01(0.04)	0.015
Participating organizations									0.000
Yes	48(13.48)	61(12.58)	0.699	109(12.96)	100(29.15)	76(27.54)	0.657	176(28.43)	
No	308(86.52)	424(87.42)		732(87.04)	243(70.85)	200(72.46)		443(71.57)	
Trusting people									0.882
Yes	217(61.30)	258(53.75)	0.030	475(56.95)	206(60.41)	143(51.81)	0.032	349(56.56)	
No	137(38.70)	222(46.25)		359(43.05)	135(39.59)	133(48.19)		268(43.44)	
**Subjective attitude**									
Life satisfaction	4.09(0.05)	3.52(0.05)	0.000	3.76(0.04)	4.14(0.05)	3.55(0.07)	0.000	3.88(0.04)	0.030
Having trust in the future	3.88(0.06)	3.27(0.06)	0.000	3.53(0.04)	3.97(0.06)	3.36(0.07)	0.000	3.70(0.05)	0.004

### Detecting potential predictors

In Table 2, we first performed a series of Chi-square and T-test analyses to examine the difference between rural and urban variables (*P* value_1_). Then, we used a series of Chi-square and T-test analyses to test the difference between depressive and non-depressive variables of urban and rural disabled elderly, respectively, *P* < 0.1 was included in the RFC. If P < 0.1, we included this variable in the subsequent RFC model.

Therefore, the input variables (features) in the rural groups were classified as follows: age, gender, marital status, education, family per capita income, drinking more than three times a week, sleep duration, regular exercise, BMI, disease, or accidence experience within the past 2 weeks, chronic disease, hospitalization within 1 year, total medical expenses within in 1 year, self-rated health, changing in perceived health, receiving financial assistance from children, weekly family dinner, neighborhood help, neighborhood relationship, community emotion, life satisfaction, having trust in the future, and trusting people.

The input variables (features) in the urban groups were classified as follows: family per capita income, drinking more than three times a week, sleep duration, regular exercise, BMI, disease or accidence experience within the past 2 weeks, chronic disease, hospitalization within 1 year, total medical expenses within 1 year, self-rated health, changing in perceived health, weekly family dinner, neighborhood help, neighborly relations, community emotion, life satisfaction, having trust in the future, trusting people, and closing to children.

### Testing prediction accuracy of potential predictors

**Fig. 2:**
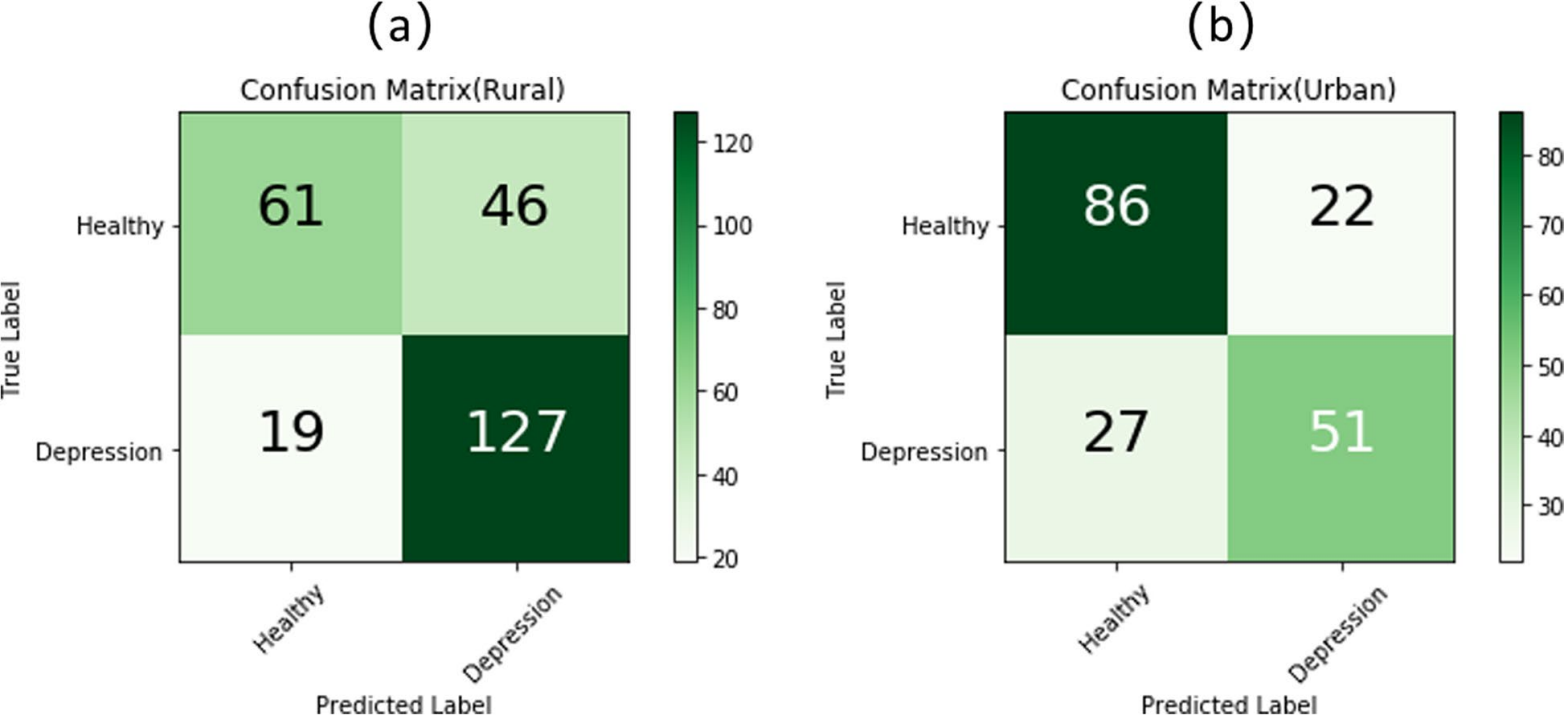
**a** Confusion matrices for rural disabled elderly **b** Confusion matrices for urban disabled elderly

The total sample was divided into two sub-samples for the analysis with a random forest algorithm: one train set and one test set. Figure 2 shows the test set confusion matrices for rural disabled elderly and urban disabled elderly. Based on previous studies [21], the other two dimensions’ accuracy, sensitivity, and specificity were also calculated.

Sensitivity refers to the true positive rate, which is calculated as follows:

$$\mathrm{Sensitivity}=\mathrm{TP}\ \left(\mathrm{True}\ \mathrm{Positive}\right)/\left(\mathrm{TP}\ \left(\mathrm{True}\ \mathrm{Positive}\right)+\mathrm{FN}\ \left(\mathrm{False}\ \mathrm{Negative}\right)\right)$$

This study refers to the proportion of disabled older adults with depression who are correctly predicted. The sensitivity score for rural disabled elderly was 80.6%, and that of urban elderly was 64.2%.

Specificity is the true negative rate, which is calculated with the following formula:

$$\mathrm{Specificity}=\mathrm{TN}\ \left(\mathrm{True}\ \mathrm{Negative}\right)/\left(\mathrm{TN}\ \left(\mathrm{True}\ \mathrm{Negative}\right)+\mathrm{FP}\ \left(\mathrm{False}\ \mathrm{Positive}\right)\right)$$

The specificity score for rural disabled elderly was 65.3%, and that of urban elderly was 78.1%.

Then, the classifier’s performance was tested by the 10-k cross-validated method, and the result was 0.71 for rural areas and 0.70 for urban areas.

**Fig. 3:**
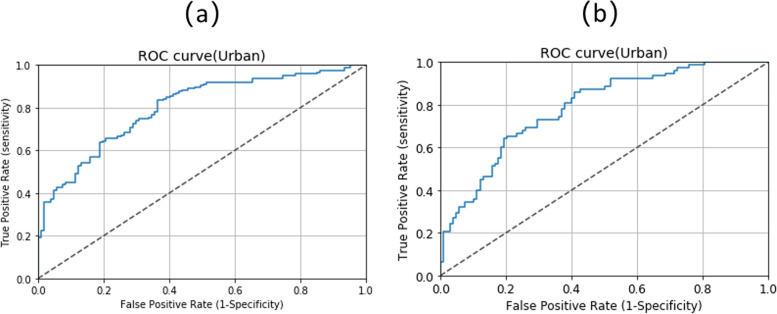
ROC Curves for depression **a** Rural **b** Urban

The typical characteristic of the receiver operating characteristic (ROC) curve is that Y-axis is the true positive rate, and X-axis is the false positive rate. The upper left corner of the graph is an ideal point to indicate that the false positive rate is 0 and the true positive rate is 1. A larger area under the curve (AUC) is usually better. The AUC of rural disabled elderly and urban disabled elderly were 0.7905 (see Fig. 3a) and 0.7781 (see Fig. 3b).

### Feature importance

**Fig. 4:**
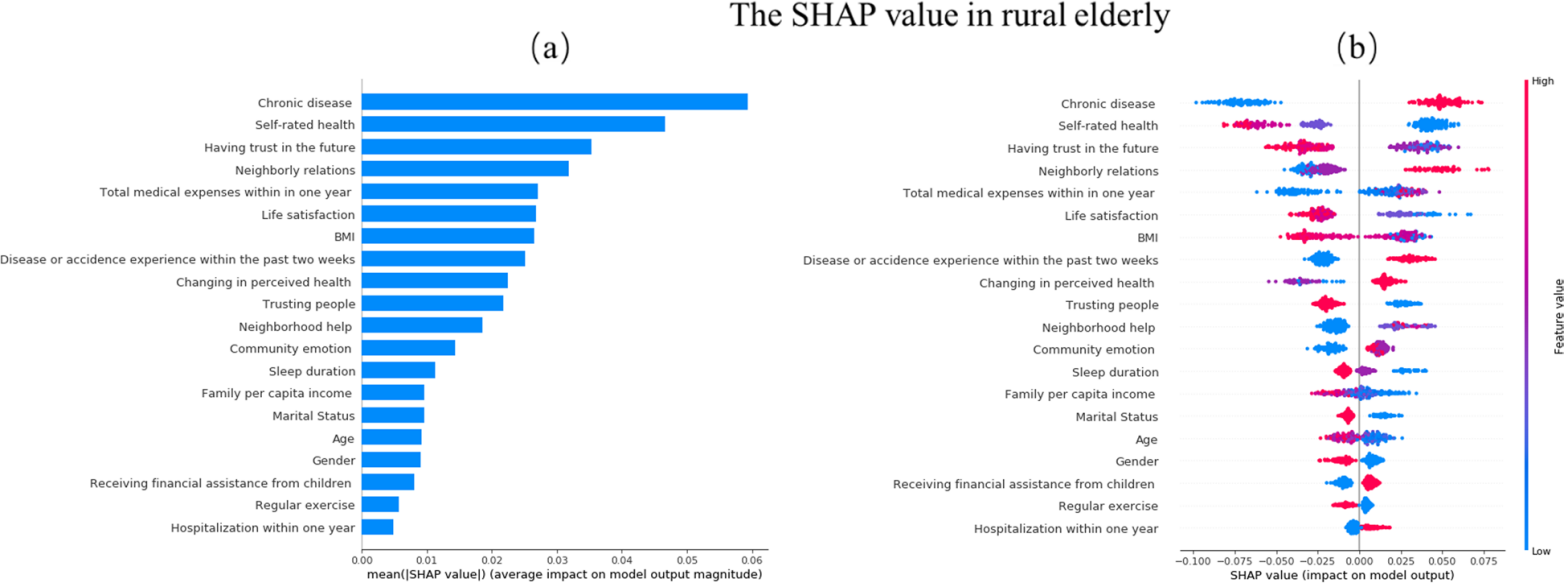
Feature importance in Random Forest Classifier. **a** Mean SHAP value **b** SHAP value(Rural)

**Fig. 5:**
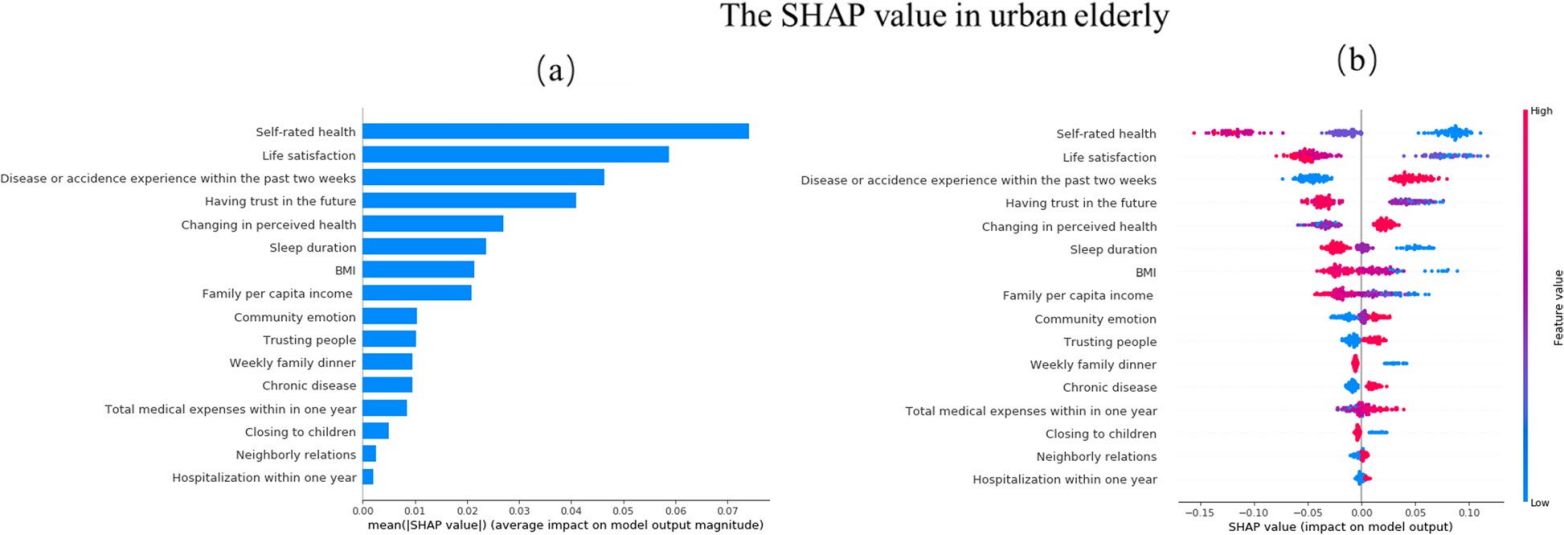
Feature importance in Random Forest Classifier. **a** Mean SHAP value **b** SHAP value(Urban)

In constructing the classification model, it is important to introduce the local interpretable technique, SHAP value calculation, and evaluation to explain the model’s data results. Figures 4 and 5 show the importance of the features evaluated by each model in descending order. The y-axis represents the features of the evaluation. The color represents the height of the feature value: the farther the distance between the points on the x-axis, the greater the influence of the feature on depression prediction.

Red means the characteristic value is relatively high, and blue means that the characteristic value is relatively low. The more right the shap value is, the greater the positive contribution to the prediction of depression. In contrast, the more left, the smaller the shap value is, the greater the negative contribution to the prediction of depression. If the shap value can distinguish between red and blue, it can be proved that their high or low values have different effects on the final results.

From Fig. 4a, the top 10 predictors for rural disabled elderly were: chronic disease, self-rated health, having trust in the future, neighborly relations, total medical expenses within 1 year, life satisfaction, BMI, disease or accidence experience within the past 2 weeks, changing in perceived health, and trusting people.

Figure 4b shows without chronic disease, better self-rated health, more confidence in the future, better neighborhood relationship, lower total medical expenses within 1 year, higher life satisfaction, higher BMI, without disease or accidence experience within the past 2 weeks, perceived health better or unchanged and deeper trust in people have greater negative contributions to depression for rural disabled elderly.

From Fig. 5a, The top 10 predictors for urban disabled elderly were: self-rated health, life satisfaction, disease or accidence experience within the past 2 weeks, having trust in the future, changing in perceived health, sleep duration, BMI, family per capita income, community emotion, and trusting people.

Figure 5b shows better self-rated health, higher life satisfaction, without disease or accidence experience within the past 2 weeks, more confidence in the future, perceived health better or unchanged, longer sleep duration, higher BMI, higher family per capita income, deeper community emotion and deeper trust in people have greater negative contributions to depression for urban disabled elderly.

## Discussion

In this study, we built a machine learning-based predictive model for rural and urban disabled older people. The depression rate of rural disabled elderly was 57.67%, higher than that of urban disabled elderly (44.59%). The mean value of the 10-k cross-validated results was 0.71 in rural areas and 0.70 in urban areas. Moreover, the AUC, specificity, and sensitivity scores of rural disabled elderly were 0.79, 65.3, and 80.6%. In contrast, urban disabled elderly were 0.78, 78.1, and 64.2%, respectively. The above result shows that this model could be practically used to screen rural and urban disabled elderly people prone to depression. There are some studies using machine learning to predict depression.

Zhang predicted depression among pregnant women through Weill Cornell Medical (WCM) data, and the AUC of their model was 0.937(development datasets) and 0.886(validation datasets). They used indicators including clinical features related to mental health history, medical comorbidity, obstetric complications, medication prescription orders, and patient demographic characteristics [22]. Dinga predicted the naturalistic course of depression from a wide range of clinical, psychological, and biological data, and their AUC values ranged from 0.66 to 0.69 [23]. Other scholars use data without clinical symptoms or indicators. Gokten and Uyulan used sociodemographic data and characteristics of sexual abuse to predict the development of depression in sexually abused children, and their accuracy of the study was 72.0% [14]. Although scholars all used the machine learning method, prediction accuracy was discrepant. We think it may relate to the predictors, research objects, and sample sources.

The ability of machine learning to detect key features from complex data sets reveals its importance. Our study found differences in the top ten predictors between the rural and urban disabled elderly. For the rural elderly, the most important feature of the random forest classifier is a chronic disease. However, chronic diseases are not the top ten predictors for urban elderly. This difference may be due to the difference in medical timeliness caused by urban and rural socioeconomic status and geographical location, which leads to the different severity of chronic diseases [24–27]. For some chronic diseases, such as diabetes, heart disease, and cerebrovascular disease, the treatment of these diseases depends on the economic status of rural residents. In contrast, urban residents would visit the doctor if they had any disease [28]. Social relationship is also an important factor in preventing and improving depression [29, 30]. Older adults exhibited better mental health in neighborhoods where positive neighborly interactions prevailed over individual adversities [31]. Our study found that the prediction effect of neighborhood relationships is more obvious among the disabled elderly in rural areas. The possible reason may be that social ties appear more consequential for attachment in rural people than in urban areas, and there are differences in social cohesion between rural and urban areas [32–34].

The prevalence of depression varied substantially between urban and rural older adults, and the prevalence of depression of urban, rural, and all older adults were 44.59, 57.67, and 52.12%, respectively. Overall, the depression rate of the disabled in the study was lower than that in the Özlem and Ünsal study (57.8%) [8]. The most likely reason may be the difference in disability measurement. Our study used IADL to measure disability, while the Özlem and Ünsal studies were based on data provided by disabled individuals registered in the Turkish Disabled Association Branch. He found the prevalence of baseline depression symptoms was 29.5, 58.0, and 73.6% in subjects with basic ADL scores of 0, 1, and ≥ 2, respectively, which shows differences in depression rates due to differences in different measurement methods of disability [23].

For the important factors to predict depression in both rural disabled elderly and urban disabled elderly, health status is one of the most important predictors of depression, including self-rated health, without disease or accidence experience within the past 2 weeks, changing in perceived health, and BMI. Self-rated health is a significant predictor, mainly because it is a multidimensional structure, including physiological, psychological, functional, and social variables. Although self-rated health is commonly seen as a manifestation of depressed affect, it seems to predict the subsequent mental health results [35]. Previous scholars have found that depression is closely related to self-rated health [36–38]. Higher BMI causes lower depressive symptoms, which the “jolly fat” hypothesis can explain. Obese people may be happier because they may not be exposed to strict diets that lead to depression [39]. Meanwhile, life satisfaction and having trust in the future were the other significant predictors of depression [40]. We were surprised to find a close relationship between trusting people and depression among the elderly in rural and urban areas. Trust itself has been shown to be associated with a host of health outcomes [41, 42].

From our research, this could be good news for the rural elderly, who have limited access to good health facilities. Using only risk factors for depression prediction means that depression can be valued even before symptoms appear, which will lead to early intervention. However, the present study has two limitations. First, due to the limitation of CFPS investigation content, our model did not include clinical symptoms or indicators. The previous research has shown that clinical symptoms or indicators contribute to the pathophysiology of depression [43–45], but our research lacks this information. Second, we did not assess the severity of depression in the rural disabled elderly.

## Conclusion

We suggest that the depression of the disabled elderly can be predicted by machine learning method from six aspects: demographic characteristics, health status, health behavior, family relationship, social relationship, and subjective attitude. There are differences in the top ten predictors between the rural and urban disabled elderly. However, we should further consider the clinical symptoms or indicators in future research.

Using random forest data to predict the depression of the disabled elderly can detect the depression early. The prediction model of this study could provide support for the intervention of depression risk identification of rural and urban disabled elderly and improve their health status through early prevention, diagnosis, and treatment.

## Data Availability

Publicly available datasets were analyzed in this study. This data can be found here: China Family Panel Studies. (http://www.isss.pku.edu.cn/cfps/).

## Abbreviations

RF: Random Forest
CES-D: Centre Epidemiological Studies Depression Scale
BMI: Body Mass Index
TP: True Positive
FN: False Negative
FP: False Positive
AUC: Area Under the Curve
ROC: Receiver Operating Characteristic
